# Prognostic Value of Subependymal Enhancement on Baseline MRI in Primary Central Nervous System Lymphoma

**DOI:** 10.3390/cancers18182906

**Published:** 2026-09-08

**Authors:** Dongsub Kim, Dong-Sup Chung, Wan-Soo Yoon

**Affiliations:** Department of Neurosurgery, Incheon St. Mary’s Hospital, College of Medicine, The Catholic University of Korea, Seoul 21431, Republic of Korea; jjeobii@gmail.com (D.K.); dschung@catholic.ac.kr (D.-S.C.)

**Keywords:** primary central nervous system, lymphoma, magnetic resonance imaging, chemoradiotherapy, prognosis

## Abstract

**Background/Objectives:** Baseline magnetic resonance imaging (MRI) findings may provide prognostic information beyond existing clinical scoring systems in primary central nervous system lymphoma (PCNSL). This study evaluated whether baseline MRI findings predict survival in immunocompetent PCNSL and assessed the clinical implications of subependymal enhancement for treatment decision-making. **Methods:** Fifty immunocompetent PCNSL patients treated at a single institution (2010–2024) were retrospectively analyzed. Baseline MRI features, including subependymal enhancement, leptomeningeal spread, and ventricular involvement, were evaluated. Univariate and multivariate Cox analyses were performed for progression-free survival (PFS) and overall survival (OS), and the relationship between subependymal enhancement and chemoradiotherapy outcome was assessed by log-rank and interaction testing. **Results:** Median PFS and OS were 21.1 and 23.6 months, respectively. On multivariate analysis, subependymal enhancement was the strongest independent predictor of both PFS (hazard ratio [HR] 2.447, 95% confidence interval [CI] 1.164–5.145; *p* = 0.018) and OS (HR 2.607, 95% CI 1.279–5.313; *p* = 0.008), and leptomeningeal spread was independently associated with worse OS (HR 3.418, 95% CI 1.473–7.932; *p* = 0.004). Among patients with subependymal enhancement, chemoradiotherapy was associated with markedly improved PFS (33.5 vs. 3.0 months; *p* = 0.029) and OS (39.8 vs. 13.4 months; *p* = 0.029) and a higher complete response rate (100% vs. 0%) compared with chemotherapy alone. No significant chemoradiotherapy benefit was observed in patients without subependymal enhancement (PFS *p* = 0.332; OS *p* = 0.907). **Conclusions:** Subependymal enhancement and leptomeningeal spread independently predict survival in PCNSL. Although subependymal enhancement marks a poor-risk subgroup overall, patients with this feature who received chemoradiotherapy achieved markedly better outcomes, suggesting a role for early chemoradiotherapy in this population. Systematic MRI assessment at diagnosis may guide individualized treatment decisions beyond existing clinical scoring systems.

## Simple Summary

Primary central nervous system lymphoma is a rare, aggressive brain cancer. Doctors currently estimate a patient’s prognosis using only clinical and blood test information, without considering findings from the brain MRI scan obtained at diagnosis. In this study of 50 patients treated over 15 years, we examined whether two MRI patterns, called subependymal enhancement and leptomeningeal spread, could help predict survival. Both features were independently linked to worse survival. Importantly, patients with subependymal enhancement who received combined chemotherapy and radiotherapy lived substantially longer than those treated with chemotherapy alone, whereas adding radiotherapy made no difference for patients without this feature. These findings suggest that a routine baseline MRI scan could help identify which patients are most likely to benefit from adding radiotherapy to their treatment plan, potentially improving outcomes in this difficult-to-treat cancer.

## 1. Introduction

Primary central nervous system lymphoma (PCNSL) is a rare and aggressive subtype of extranodal non-Hodgkin lymphoma confined to the brain, spinal cord, cerebrospinal fluid (CSF), and eyes, without evidence of systemic disease at diagnosis [1,2]. Histologically, the vast majority of cases are classified as diffuse large B-cell lymphoma (DLBCL) and the disease accounts for approximately 1–5% of all primary brain tumors [2,3]. The estimated annual incidence is approximately 0.4–0.5 per 100,000 population, with a marked age-related gradient (approximately 0.08 per 100,000 among patients aged 20–29 years versus 4.32 per 100,000 among those aged 70–79 years) and a rising incidence among older adults over the past four decades [2,4]. At the molecular level, PCNSL is characterized by recurrent activating mutations in genes regulating the B-cell receptor and Toll-like receptor signaling pathways—most notably *MYD88* (present in approximately 70–80% of cases, predominantly the L265P variant) and *CD79B*—which converge on constitutive activation of nuclear factor kappa B (NF-κB) and promote tumor cell proliferation while impairing apoptosis; these pathway alterations have informed the development of targeted agents such as Bruton’s tyrosine kinase (BTK) inhibitors [5,6]. The introduction of high-dose methotrexate (HD-MTX)-based chemotherapy have substantially improved outcomes over whole-brain radiotherapy (WBRT) alone [7,8]. Nevertheless, prognosis remains poor, with 5-year overall survival (OS) rates of only 30–40% in unselected populations [1,3,4], and a significant risk of treatment-related neurotoxicity [9,10].

Risk stratification in PCNSL currently relies on the International Extranodal Lymphoma Study Group (IELSG) prognostic score and the Memorial Sloan-Kettering Cancer Center (MSKCC) recursive partitioning model [11,12]. However, both systems rely exclusively on clinical and laboratory parameters, without incorporating baseline magnetic resonance imaging (MRI) findings, which are routinely obtained at diagnosis and may provide independent, immediately available, and clinically actionable prognostic information at the time of treatment planning [13,14,15]. Furthermore, the involvement of deep structures of the brain criterion in the IELSG score subsumes a heterogeneous spectrum of tumor locations under a single binary variable, potentially limiting its prognostic precision [11,16].

Among the MRI features observed in PCNSL, subependymal enhancement, defined as linear or curvilinear contrast enhancement along the ependymal lining of the ventricular walls, and leptomeningeal spread represent distinct patterns of tumor dissemination that have been described in CNS lymphoma [13,17] but have not been systematically characterized as prognostic biomarkers in immunocompetent PCNSL. In the present study, we retrospectively analyzed 50 immunocompetent PCNSL patients treated over 15 years to evaluate whether these baseline MRI findings independently predict survival outcomes, and to assess whether subependymal enhancement may further identify patients who derive substantial survival benefit from the inclusion of chemoradiotherapy in their first-line treatment regimen.

## 2. Materials and Methods

### 2.1. Study Design and Patients

This was a single-center retrospective cohort study. We identified consecutive patients diagnosed with PCNSL between January 2010 and December 2024 at our hospital. Inclusion criteria were: (1) histologically confirmed PCNSL (DLBCL type); (2) age ≥ 18 years; (3) absence of systemic lymphoma at diagnosis; and (4) availability of baseline MRI. Patients with human immunodeficiency virus infection, prior immunosuppressive therapy, or secondary CNS lymphoma were excluded. A total of 50 patients were included. This study was approved by the Institutional Review Board of Incheon St. Mary’s Hospital, the Catholic University of Korea (IRB No. OC26RID10143), and informed consent was waived due to the retrospective design. As this was a retrospective analysis of all consecutive eligible patients treated during the study period rather than a prospectively powered study, no a priori sample size calculation was performed.

### 2.2. Clinical Data Collection

Baseline clinical characteristics included age, sex, Karnofsky Performance Status (KPS), Eastern Cooperative Oncology Group performance status (ECOG PS), and serum lactate dehydrogenase (LDH). Surgical approach was categorized as stereotactic biopsy, open biopsy, or craniotomy. First-line treatment regimens were classified as HD-MTX alone, ifosfamide-carboplatin-etoposide with or without radiotherapy (ICE ± RT), methotrexate-procarbazine-vincristine with or without radiotherapy (MPV ± RT), or RT alone. Chemoradiotherapy was defined as chemotherapy followed by planned RT as part of the first-line regimen (ICE + RT or MPV + RT). HD-MTX was administered at 8.0 g/m^2^ in most patients (28/30), with a reduced dose (3.5–4.0 g/m^2^) used in two patients. Among patients receiving ICE or MPV, the number of chemotherapy cycles ranged from 1 to 6. Among the 15 patients who received radiotherapy (13 as chemoradiotherapy and 2 as RT alone), WBRT was delivered with or without a consolidative boost, with a total RT dose ranging from 23.4 to 50.6 Gy in 13–35 fractions; full per-patient chemotherapy and radiotherapy details are provided in Supplementary Table S1. Radiologic response was assessed per the International PCNSL Collaborative Group criteria and categorized as complete response (CR), partial response (PR), stable disease (SD), or progressive disease (PD).

### 2.3. MRI Assessment

**Figure 1:**
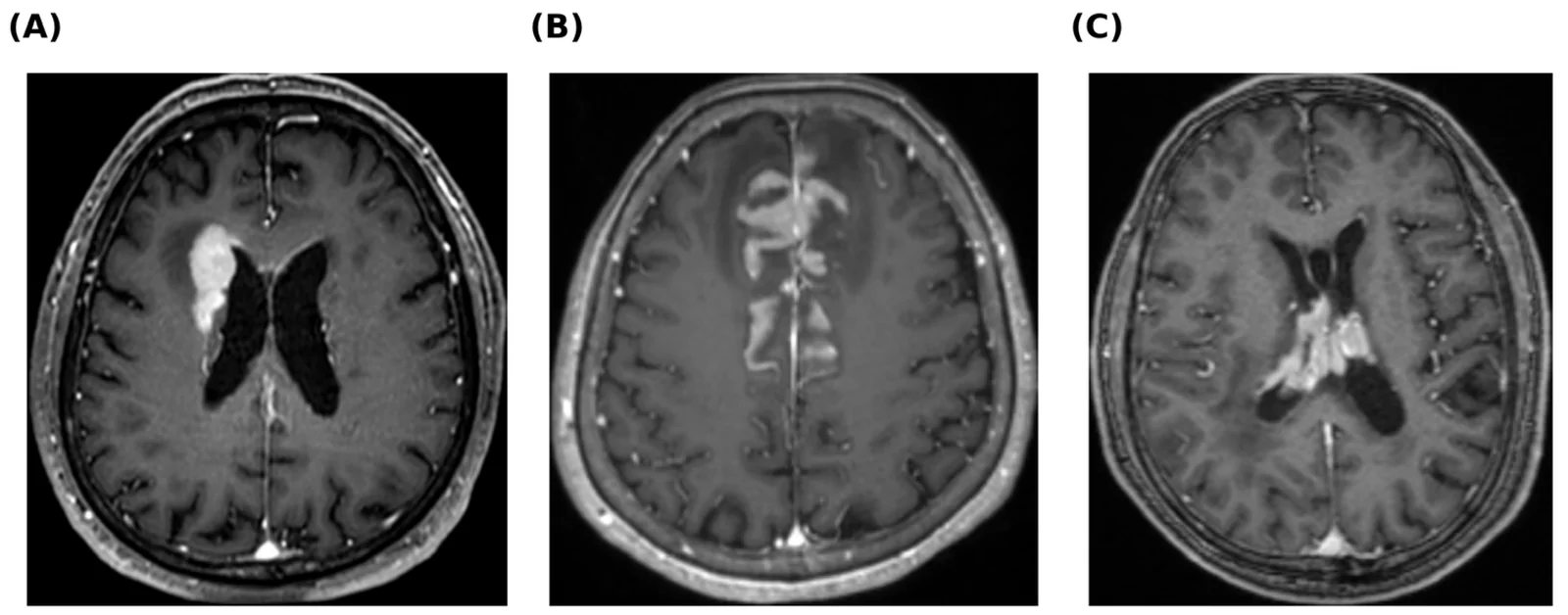
Representative magnetic resonance imaging (MRI) findings in primary central nervous system lymphoma. Axial contrast-enhanced T1-weighted MR images. (**A**) Curvilinear contrast enhancement along the right lateral ventricular wall, consistent with subependymal enhancement. (**B**) Linear enhancement along the leptomeningeal surfaces of the bilateral frontal lobes, consistent with leptomeningeal spread. (**C**) Bilateral enhancing masses within the lateral ventricular cavities, consistent with ventricular involvement.

Baseline brain MRI was reviewed by a neuroradiologist blinded to clinical outcomes. The following features were evaluated: (1) number of lesions (solitary vs. multiple); (2) radiologic location (cortical vs. subcortical/deep structures); (3) subependymal enhancement, defined as linear or curvilinear contrast enhancement along the ventricular wall (ependymal lining) (Figure 1A); (4) leptomeningeal spread, defined as linear or nodular enhancement along leptomeningeal surfaces, cranial nerves, or an enhancing mass occupying the cisternal space without identifiable parenchymal origin (Figure 1B); and (5) ventricular involvement, defined as direct tumor extension into the ventricular cavity (Figure 1C).

### 2.4. Definition of Survival Outcomes

Progression-free survival (PFS) was defined as the time from initiation of first-line treatment to confirmed disease recurrence on MRI. Patients without confirmed recurrence (no recurrence or unknown status) were treated as true missing for the PFS event definition and were censored at their OS time. OS was defined as the time from diagnosis to death from any cause; surviving patients were censored at the last follow-up date. As a sensitivity analysis addressing potential informative censoring among patients with unconfirmed recurrence, PFS was additionally analyzed using a composite progression-or-death endpoint, in which the event was defined as confirmed recurrence or death from any cause, whichever occurred first; patients with neither event were censored at last follow-up.

### 2.5. Statistical Analysis

Categorical variables are presented as frequencies and percentages, and continuous variables as median with range. Survival curves were estimated using the Kaplan–Meier method and compared between groups using the log-rank test. Univariate Cox proportional hazards regression was performed to estimate hazard ratios (HRs) with 95% confidence intervals (CIs) for each prognostic variable. Given the small sample size, a parsimonious multivariate model was constructed using three variables selected based on clinical relevance and univariate analysis results: subependymal enhancement, leptomeningeal spread, and chemoradiotherapy. To evaluate the interaction between subependymal enhancement and chemoradiotherapy in first-line treatment, a Cox model including both variables and their product term (subependymal enhancement × chemoradiotherapy) was fitted; the likelihood ratio test was used to assess the statistical significance of the interaction term. Stratified log-rank tests were further performed within subependymal enhancement subgroups (Yes vs. No) to assess the chemoradiotherapy effect in each stratum. Treatment response rates (CR and overall response rate [ORR; CR + PR]) were compared between subgroups using Fisher’s exact test. Non-parametric comparisons of continuous variables were performed using the Mann–Whitney U test. To assess whether the prognostic value of subependymal enhancement was independent of established clinical risk models, the MSKCC recursive partitioning class was calculated for each patient using age and KPS. Because CSF protein data were not uniformly available in this retrospective cohort, a partial IELSG score was calculated using the four available adverse factors (age > 60 years, ECOG PS > 1, elevated LDH, and involvement of deep brain structures); this partial score, which omits CSF protein, should be interpreted with caution. Model discrimination with and without subependymal enhancement was compared using Harrell’s concordance index. Because chemoradiotherapy was not administered to any patient treated before 2018, an additional multivariate model incorporating treatment era (2010–2017 vs. 2018–2024) as a covariate was fitted to evaluate whether the observed associations were confounded by temporal changes in practice. Baseline characteristics were also compared across the four subependymal enhancement/chemoradiotherapy subgroups (Groups A–D; see Section 3.5) using Fisher’s exact test and the Mann–Whitney U test to assess potential confounding by indication. All statistical tests were two-sided, and *p* < 0.05 was considered statistically significant. Statistical analyses were performed using Python (version 3.12; Python Software Foundation, Wilmington, DE, USA) with the lifelines (version 0.27) and SciPy (version 1.11.0; NumFOCUS, Austin, TX, USA) libraries.

## 3. Results

### 3.1. Patient Characteristics

A total of 50 patients with PCNSL were included in this study. The median age was 63 years (range, 33–79 years), and 32 patients (64.0%) were older than 60 years. The male-to-female ratio was 28:22. The median KPS at diagnosis was 70 (range, 30–90), with 29 patients (58.0%) having 70 or greater. ECOG PS was 0–1 in 14 (28.0%), 2 in 21 (42.0%), and 3–4 in 15 (30.0%) patients. Serum LDH was evaluable in 40 patients; of these, 15 (37.5%) had elevated LDH.

On baseline MRI, solitary and multiple lesions were equally distributed. Subcortical or deep structure location was identified in 23 patients (46.0%), subependymal enhancement in 22 patients (44.0%), ventricular involvement in 9 (18.0%), and leptomeningeal spread in 9 (18.0%). Stereotactic biopsy was performed in 15 patients (30.0%), open biopsy in 24 (48.0%), and craniotomy in 11 (22.0%; non-gross total resection [GTR] in 5, GTR in 6). Chemoradiotherapy was not used in any patient treated before 2018; among patients treated in 2010–2017 (*n* = 22), all received HD-MTX alone, whereas among those treated in 2018–2024 (*n* = 28), 13 (46.4%) received chemoradiotherapy, reflecting a temporal shift in treatment practice over the 15-year study period (see Section 3.6 and Section 4).

Regarding first-line treatment, 30 patients (60.0%) received HD-MTX alone, 8 (16.0%) received ICE ± RT, 10 (20.0%) received MPV ± RT, and 2 (4.0%) received radiotherapy alone. Of these, 13 patients (26.0%) received chemoradiotherapy (ICE + RT or MPV + RT) as part of their first-line regimen. Radiologic response after the first-line treatment was assessed in all 50 patients; response was not evaluable in 6 (12.0%) because of death or loss to follow-up before imaging reassessment. Among the entire cohort (*n* = 50, as shown in Table 1), CR was achieved in 25 (50.0%), PR in 6 (12.0%), SD in 1 (2.0%), and PD in 12 (24.0%). Among the 44 response-evaluable patients, CR was achieved in 25 (56.8%), PR in 6 (13.6%), SD in 1 (2.3%), and PD in 12 (27.3%). The ORR was 62.0% (31/50) among all patients and 70.5% (31/44) among response-evaluable patients.

### 3.2. Survival Outcomes

**Figure 2:**
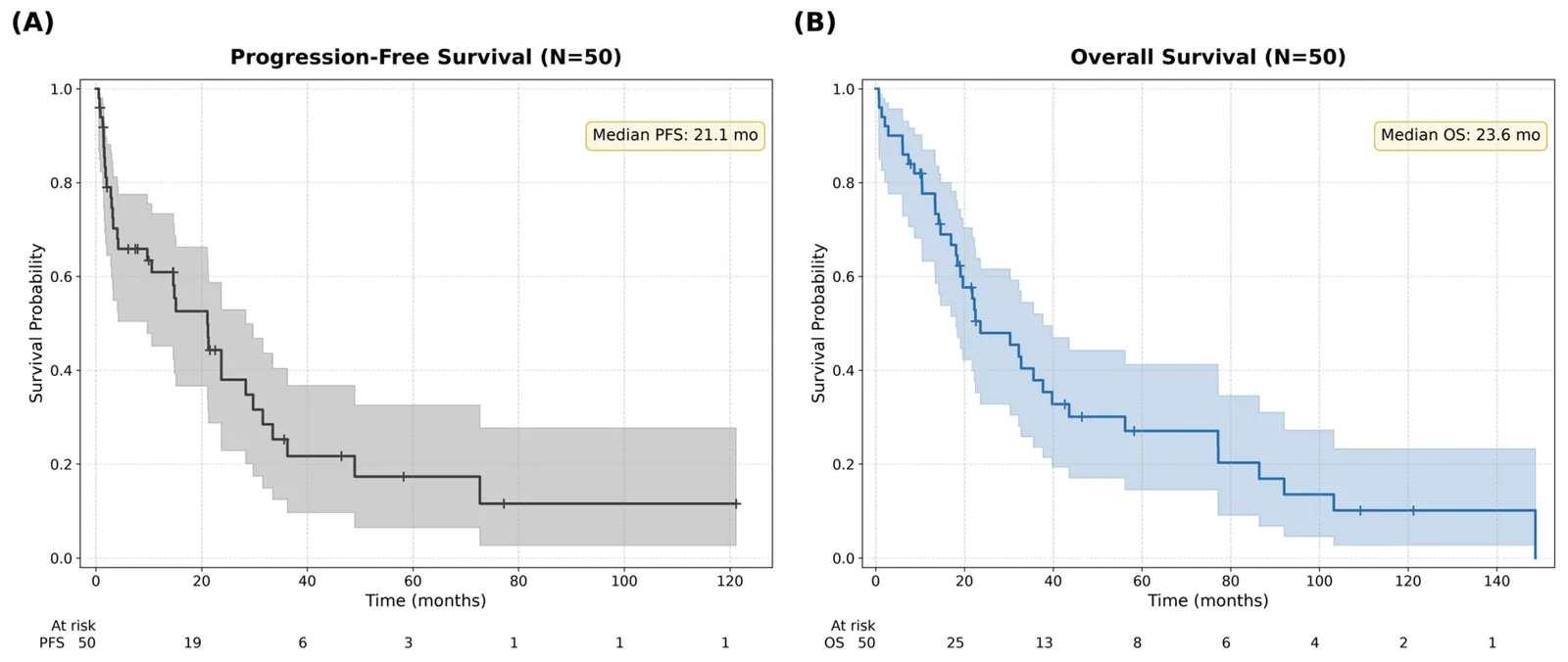
Kaplan–Meier survival curves for the entire cohort (*n* = 50). (**A**) Progression-free survival (PFS). Median PFS was 21.1 months (33 events, 17 censored); PFS rates were 65.8%, 60.9%, 37.9%, and 25.3% at 6, 12, 24, and 36 months, respectively. (**B**) Overall survival (OS). Median OS was 23.6 months; OS rates were 90.0%, 77.6%, 47.9%, and 37.8% at 6, 12, 24, and 36 months, respectively. Shaded areas indicate 95% confidence intervals; tick marks indicate censored observations.

With a median follow-up of 20.6 months (range, 0.7–148.7 months), confirmed disease recurrence following first-line treatment was documented in 33 patients (66.0%), while 9 (18.0%) had no recurrence and 8 (16.0%) had an unknown recurrence status. The overall median PFS was 21.1 months (events: 33/50; censored: 17). PFS rates were 65.8% (95% CI, 50.4–77.5%) at 6 months, 60.9% (95% CI, 45.1–73.3%) at 12 months, 37.9% (95% CI, 22.9–52.9%) at 24 months, and 25.3% (95% CI, 9.7–36.7%) at 36 months (Figure 2A).

At the time of analysis, 38 patients (76.0%) had died, 11 (22.0%) were alive, and 1 (2.0%) was lost to follow-up. The median OS for the entire cohort was 23.6 months. OS rates were 90.0% (95% CI, 75.2–94.4%) at 6 months, 77.6% (95% CI, 60.8–85.3%) at 12 months, 47.9% (95% CI, 32.8–61.6%) at 24 months, and 37.8% (95% CI, 23.6–52.0%) at 36 months (Figure 2B).

### 3.3. Univariate Analysis of PFS and OS

Among all variables evaluated on univariate analysis, MRI findings and treatment modality were most strongly associated with survival outcomes. Among MRI features, subependymal enhancement showed a borderline association with shorter PFS (Yes vs. No: 3.2 vs. 23.7 months; HR = 1.792; 95% CI, 0.934–3.437; *p* = 0.054). Regarding treatment modality, when analyzed with chemoradiotherapy as the reference group, chemotherapy alone was associated with significantly shorter PFS (median 10.6 vs. 33.5 months; HR = 2.302; 95% CI, 1.047–5.060; *p* = 0.024), while radiotherapy alone showed a trend toward worse PFS without reaching statistical significance (median 3.3 months; HR = 4.653; 95% CI, 0.400–54.155; *p* = 0.074). Other clinical and imaging variables, including age, sex, KPS, ECOG PS, serum LDH, number of lesions, radiologic location, ventricular involvement, and leptomeningeal spread, were not significantly associated with PFS (all *p* > 0.10) (Table 2).

For OS, the two MRI features of primary interest, subependymal enhancement and leptomeningeal spread, were identified as significant prognostic factors. The presence of subependymal enhancement was significantly associated with shorter OS (median 17.0 vs. 37.7 months; HR = 2.075; 95% CI, 1.089–3.954; *p* = 0.014), and leptomeningeal spread was associated with markedly worse OS (median 13.4 vs. 32.2 months; HR = 2.572; 95% CI, 1.174–5.635; *p* = 0.007). With respect to treatment, chemoradiotherapy did not show a statistically significant association with OS compared with chemotherapy alone (median 43.6 vs. 21.8 months; HR = 0.547; 95% CI, 0.223–1.343; *p* = 0.182). Although RT alone showed a marginal *p*-value for worse OS compared with chemoradiotherapy (*p* = 0.047), this finding is not interpretable given the extremely small sample size (*n* = 2) and an extremely wide CI (HR = 4.415; 95% CI, 0.713–27.355). All remaining clinical and imaging variables were not significantly associated with OS (all *p* > 0.10) (Table 2).

Subependymal enhancement was also significantly associated with treatment response. The CR rate was markedly lower in patients with subependymal enhancement than in those without (18.2% vs. 75.0%, *p* < 0.001), and the ORRwas similarly lower (31.8% vs. 85.7%, *p* < 0.001), indicating that the presence of subependymal enhancement on baseline MRI was strongly associated with a poor response to first-line chemotherapy-based treatment.

### 3.4. Multivariate Analysis of PFS and OS

Multivariate Cox proportional hazards analysis was performed using three variables: subependymal enhancement and leptomeningeal spread, selected as the primary MRI-based prognostic factors of interest identified on univariate analysis, and chemoradiotherapy, selected as the treatment variable given its stronger and more clinically homogeneous association with survival (Table 3).

For PFS, subependymal enhancement and chemoradiotherapy were identified as independent prognostic factors. Subependymal enhancement was independently associated with a significantly higher risk of disease recurrence (HR = 2.447; 95% CI, 1.164–5.145; *p* = 0.018), while chemoradiotherapy was independently associated with a significantly lower risk of recurrence (HR = 0.293; 95% CI, 0.115–0.741; *p* = 0.010). Leptomeningeal spread was not independently associated with PFS on multivariate analysis (HR = 1.427; 95% CI, 0.503–4.051; *p* = 0.504). The model demonstrated good discriminative ability (concordance index 0.725; likelihood ratio test *p* = 0.006).

For OS, subependymal enhancement and leptomeningeal spread were independently associated with significantly worse survival. Subependymal enhancement was independently associated with a significantly higher risk of death (HR = 2.607; 95% CI, 1.279–5.313; *p* = 0.008), and leptomeningeal spread was independently associated with significantly worse OS (HR = 3.418; 95% CI, 1.473–7.932; *p* = 0.004). Chemoradiotherapy showed a borderline association with improved OS that did not reach statistical significance (HR = 0.431; 95% CI, 0.174–1.071; *p* = 0.070). The model demonstrated acceptable discriminative ability (concordance index 0.672; likelihood ratio test *p* = 0.001).

### 3.5. Differential Chemoradiotherapy Effect According to Subependymal Enhancement Status

**Figure 3:**
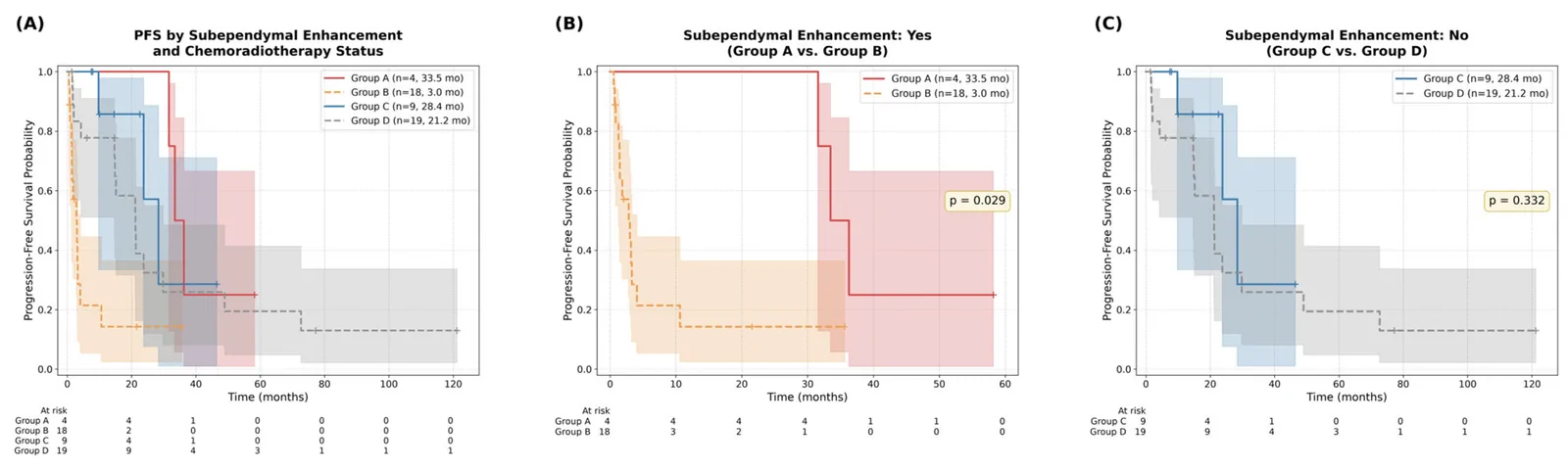
Progression-free survival (PFS) according to subependymal enhancement and chemoradiotherapy status. Patients were divided into four groups according to the presence or absence of subependymal enhancement on baseline MRI and receipt of chemoradiotherapy (chemotherapy followed by planned radiotherapy, ICE + RT or MPV + RT) as part of first-line treatment: subependymal enhancement present with chemoradiotherapy (Group A, *n* = 4); subependymal enhancement present without chemoradiotherapy (Group B, *n* = 18); subependymal enhancement absent with chemoradiotherapy (Group C, *n* = 9); and subependymal enhancement absent without chemoradiotherapy (Group D, *n* = 19). (**A**) PFS in all four groups. Median PFS was 33.5, 3.0, 28.4, and 21.2 months for Groups A, B, C, and D, respectively. (**B**) Among patients with subependymal enhancement, PFS was markedly longer in patients who received chemoradiotherapy (Group A) than in those who did not (Group B) (33.5 vs. 3.0 months; *p* = 0.029). (**C**) Among patients without subependymal enhancement, PFS did not differ significantly according to chemoradiotherapy status (Group C vs. Group D) (28.4 vs. 21.2 months; *p* = 0.332). Taken together, the PFS benefit of chemoradiotherapy was confined to patients with subependymal enhancement. Shaded areas indicate 95% confidence intervals; tick marks indicate censored observations.

**Figure 4:**
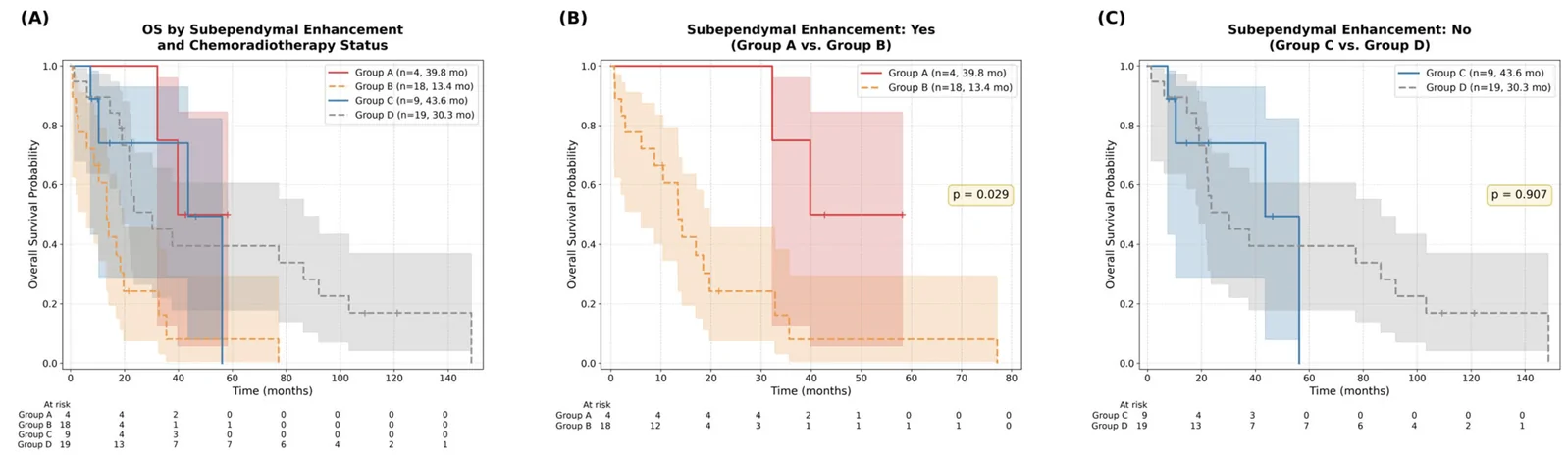
Overall survival (OS) according to subependymal enhancement and chemoradiotherapy status (groups defined as in Figure 3). (**A**) OS in all four groups. Median OS was 39.8, 13.4, 43.6, and 30.3 months for Groups A, B, C, and D, respectively. (**B**) Among patients with subependymal enhancement, OS was markedly longer in patients who received chemoradiotherapy (Group A) than in those who did not (Group B) (39.8 vs. 13.4 months; *p* = 0.029). (**C**) Among patients without subependymal enhancement, OS did not differ significantly according to chemoradiotherapy status (Group C vs. Group D) (43.6 vs. 30.3 months; *p* = 0.907). Taken together, the OS benefit of chemoradiotherapy was confined to patients with subependymal enhancement. Shaded areas indicate 95% confidence intervals; tick marks indicate censored observations.

To evaluate whether subependymal enhancement predicts differential benefit from chemoradiotherapy, patients were stratified into four subgroups according to the presence or absence of subependymal enhancement on baseline MRI and whether chemoradiotherapy was included in their first-line treatment regimen: patients with subependymal enhancement who received chemoradiotherapy (Group A, *n* = 4), patients with subependymal enhancement who did not receive chemoradiotherapy (Group B, *n* = 18), patients without subependymal enhancement who received chemoradiotherapy (Group C, *n* = 9), and patients without subependymal enhancement who did not receive chemoradiotherapy (Group D, *n* = 19) (Figure 3A and Figure 4A).

Among patients with subependymal enhancement, Group A demonstrated markedly superior outcomes compared with Group B. Median PFS was 33.5 months in Group A versus 3.0 months in Group B (*p* = 0.029) (Figure 3B), and median OS was 39.8 versus 13.4 months, respectively (*p* = 0.029) (Figure 4B). The CR rate was 100% in Group A compared with 0% in Group B (*p* < 0.001). In contrast, no statistically significant difference in PFS or OS was observed between Group C and Group D (PFS: 28.4 vs. 21.2 months, *p* = 0.332; OS: 43.6 vs. 30.3 months, *p* = 0.907) (Figure 3C and Figure 4C), suggesting that the beneficial effect of chemoradiotherapy on survival was largely confined to patients with subependymal enhancement.

The interaction between subependymal enhancement and chemoradiotherapy was formally assessed using a Cox regression model incorporating a product term. The interaction term did not reach statistical significance for either PFS (HR = 0.417; 95% CI, 0.098–1.776; *p* = 0.237) or OS (HR = 0.298; 95% CI, 0.067–1.323; *p* = 0.111), likely reflecting limited statistical power due to the small number of patients in Group A (*n* = 4). Nevertheless, the magnitude of the differential chemoradiotherapy effect across subependymal enhancement strata was clinically substantial, and the consistent findings across both PFS and OS endpoints strengthen the biological plausibility of this interaction.

Pairwise comparisons further demonstrated that Group B had significantly worse PFS and OS than those in all other subgroups. Compared with Group C, Group B showed significantly shorter PFS (3.0 vs. 28.4 months, *p* = 0.004) and a trend toward worse OS (13.4 vs. 43.6 months, *p* = 0.062). Similarly, compared with Group D, Group B had significantly worse PFS (3.0 vs. 21.2 months, *p* = 0.008) and OS (13.4 vs. 30.3 months, *p* = 0.002). Taken together, these findings suggest that subependymal enhancement on baseline MRI may identify a subset of PCNSL patients in whom chemotherapy alone is insufficient and the early incorporation of chemoradiotherapy into the first-line treatment regimen appears to confer substantial survival benefit in this exploratory subgroup analysis.

### 3.6. Sensitivity Analyses and Comparison with Existing Prognostic Models

To address potential informative censoring associated with the primary PFS definition, a sensitivity analysis was performed using a composite progression-or-death endpoint. Results were consistent with the primary analysis: subependymal enhancement remained an independent predictor of the composite endpoint on multivariate analysis (HR 2.305, 95% CI 1.187–4.477; *p* = 0.014), as did chemoradiotherapy (HR 0.288, 95% CI 0.124–0.672; *p* = 0.004). Within the subependymal enhancement subgroup, the survival advantage associated with chemoradiotherapy (Group A vs. Group B) remained significant and was, if anything, more pronounced using the composite endpoint (median 33.5 vs. 2.1 months; log-rank *p* = 0.008), whereas no significant difference was observed among patients without subependymal enhancement (Group C vs. Group D; median 28.4 vs. 15.1 months; *p* = 0.300). The formal interaction term remained non-significant (*p* = 0.113), consistent with the primary analysis.

Baseline characteristics did not differ significantly between Group A and Group B, or between Group C and Group D, with respect to age, sex, KPS, ECOG PS, or LDH (all *p* > 0.1, Fisher’s exact test or Mann–Whitney U test), providing no evidence that patients selected for chemoradiotherapy were systematically younger or fitter than those who were not; median age was, if anything, numerically higher in Group A than in Group B (71 vs. 62.5 years). Missingness of key variables was also compared across these subgroups: serum LDH was unavailable in 15.4% of chemoradiotherapy-treated versus 21.6% of non-chemoradiotherapy-treated patients (*p* = 1.000, Fisher’s exact test) and in 9.1% of subependymal enhancement-positive versus 28.6% of subependymal enhancement-negative patients (*p* = 0.154); recurrence status was unknown in 7.7% versus 18.9% of patients by chemoradiotherapy status (*p* = 0.662) and in 22.7% versus 10.7% by subependymal enhancement status (*p* = 0.277); and radiologic response was non-evaluable in 0% versus 16.2% of patients by chemoradiotherapy status (*p* = 0.319) and in 22.7% versus 3.6% by subependymal enhancement status (*p* = 0.075). None of these differences reached statistical significance, arguing against differential missingness as a source of confounding by indication.

Because chemoradiotherapy was not used in any patient treated before 2018 (0/22 in 2010–2017 vs. 13/28 [46.4%] in 2018–2024), treatment era and receipt of chemoradiotherapy were closely correlated. In a multivariate model incorporating treatment era alongside subependymal enhancement and chemoradiotherapy, subependymal enhancement remained a strong independent predictor of both PFS (HR 3.263, 95% CI 1.395–7.633; *p* = 0.006) and OS (HR 2.575, 95% CI 1.267–5.233; *p* = 0.009), while treatment era itself was not independently associated with outcome (PFS *p* = 0.209; OS *p* = 0.651). The association between chemoradiotherapy and outcome was attenuated after adjustment for era (PFS *p* = 0.062; OS *p* = 0.080), reflecting the structural overlap between chemoradiotherapy use and treatment era; the two effects could not be fully disentangled in this cohort and this limitation is discussed further below.

To evaluate whether subependymal enhancement provided prognostic information beyond established clinical risk models, the MSKCC recursive partitioning class and a partial IELSG score (four of five components, excluding CSF protein, which was not uniformly available in this retrospective cohort) were calculated for each patient. Neither the MSKCC class nor the partial IELSG score was significantly associated with OS in this cohort (*p* = 0.608 and *p* = 0.333, respectively), and model discrimination was poor for both scores alone (concordance index 0.49 for MSKCC and 0.52 for partial IELSG). Discrimination improved substantially when subependymal enhancement was added to either model (concordance index 0.61 for MSKCC plus subependymal enhancement and 0.63 for partial IELSG plus subependymal enhancement), and subependymal enhancement remained independently significant in both models (*p* = 0.015 and *p* = 0.011, respectively), supporting its incremental prognostic value beyond existing clinical scoring systems.

## 4. Discussion

PCNSL remains one of the most challenging subtypes of non-Hodgkin lymphoma [5]. Prior to HD-MTX-based chemotherapy, WBRT alone yielded a median OS of 10–18 months with rapid relapse [18,19]. HD-MTX regimens improved CR rates to 30–60% and median OS to 25–60 months [20,21,22], and the IELSG32 randomized trial established the rituximab and thiotepa to methotrexate-cytarabine as a new induction benchmark for fit patients aged up to 70 years [23,24,25]. Despite these advances, 5-year OS rates remain at 30–40% [1,4], relapse is common, and combined chemoradiotherapy carries a well-established risk of delayed neurotoxicity, particularly leukoencephalopathy, in up to 25–35% of long-term survivors [19,26]. In our cohort, median PFS and OS of 21.1 and 23.6 months, respectively, are consistent with outcomes reported in previous series [27,28].

The IELSG prognostic score and MSKCC model are the most widely validated prognostic tools in PCNSL [11,12], but both rely exclusively on clinical and laboratory variables without incorporating baseline MRI findings. The involvement of deep structures of the brain in the IELSG score, encompassing periventricular region, basal ganglia, corpus callosum, brainstem, and cerebellum under a single binary criterion, has been criticized for its broad and heterogeneous definition, which may limit its prognostic precision [11,16]. Neither system incorporates MRI-based enhancement patterns, which may provide more sensitive and specific prognostic information [13,14].

Against this background, the present study provides novel evidence that two specific baseline MRI features, subependymal enhancement and leptomeningeal spread, independently predict survival in PCNSL, offering complementary prognostic information beyond existing scoring systems. Subependymal enhancement was present in 44% of patients and emerged as the strongest independent predictor of both PFS and OS on multivariate analysis. The markedly lower CR rate in patients with subependymal enhancement compared with those without further underscores its association with a chemotherapy-resistant disease phenotype, and is consistent with the hypothesis that subependymal enhancement reflects diffuse tumor infiltration along the ependymal surface that is inadequately controlled by systemic chemotherapy alone. To the best of our knowledge, this is the first study to evaluate subependymal enhancement as an independent prognostic factor in immunocompetent PCNSL. Although subependymal enhancement has been described as a hallmark of leptomeningeal dissemination in secondary CNS lymphoma [13], its prognostic significance in immunocompetent PCNSL has not been previously investigated.

Leptomeningeal spread was independently associated with worse OS but not with PFS on multivariate analysis, revealing a dissociation between its effect on initial disease progression and long-term survival. Although leptomeningeal spread did not significantly shorten the time to first recurrence, indicating that first-line treatment achieves at least partial initial disease control even in the presence of leptomeningeal dissemination, it independently predicted a markedly higher risk of death. This pattern suggests that leptomeningeal spread identifies a subset of patients who achieve sufficient initial disease control to avoid early recurrence but are rendered highly susceptible to rapid fatal deterioration following relapse, possibly through impaired responsiveness to salvage therapy in the setting of diffuse leptomeningeal dissemination throughout the CSF compartment. Prior series have suggested a potential prognostic role of meningeal involvement in PCNSL [29,30], though this has not been consistently replicated [31,32], in part because CSF cytology has a well-recognized high false-negative rate for detecting meningeal disease [11]. MRI-based assessment of leptomeningeal enhancement may provide more sensitive and reproducible detection in this context. Clinically, identification of leptomeningeal spread at baseline MRI may alert treating physicians to patients at high risk of treatment-refractory relapse, warranting proactive discussion of salvage strategies, such as pomalidomide-based regimens for relapsed or refractory disease [33], and goals of care from the outset of treatment.

The most clinically significant finding of the present study is the marked difference in chemoradiotherapy outcome according to subependymal enhancement status. Among patients with subependymal enhancement, chemoradiotherapy was associated with markedly superior PFS, OS, and CR rate compared with chemotherapy alone. In contrast, no significant survival benefit from chemoradiotherapy was observed in patients without subependymal enhancement, in whom chemotherapy alone showed comparable efficacy. These findings suggest that the benefit of radiotherapy in PCNSL is not uniformly distributed across all patients but is concentrated in those with subependymal enhancement, a subgroup poorly controlled by chemotherapy alone.

The biological basis for this differential treatment response is likely rooted in the anatomical characteristics of subependymal enhancement. The ventricular ependymal surface is contiguous with the entire ventricular system and communicates directly with the subarachnoid space, providing a continuous pathway for widespread CSF dissemination [34]. Although HD-MTX achieves therapeutic CSF concentrations [20,21], drug penetration and exposure across the human CNS are spatially heterogeneous and vary by anatomical region [35], raising the possibility that cytotoxic exposure may be insufficient across the full extent of diffusely infiltrated ependymal surfaces. Because its cytotoxic effect does not depend on systemic drug delivery, WBRT may offer a relative advantage over chemotherapy in regions of the CNS where blood–brain barrier limitations restrict adequate drug penetration [36], potentially eradicating residual or chemotherapy-resistant tumor cells that escape systemic agents. Conversely, in patients without subependymal enhancement whose tumors are more focally parenchymal, HD-MTX achieves adequate tumor control without radiotherapy, which would carry unnecessary neurotoxicity risk. These observations are consistent with the G-PCNSL-SG-1 trial, which demonstrated that omitting WBRT after HD-MTX did not significantly compromise OS in the overall PCNSL population while reducing neurotoxicity [9], and suggest that subependymal enhancement may identify the specific subgroup in whom radiotherapy is not merely optional but essential for adequate disease control.

The therapeutic landscape of PCNSL continues to evolve, with BTK inhibitors, thiotepa-containing high-dose chemotherapy with autologous stem-cell rescue, and other strategies increasingly used to delay or avoid upfront radiotherapy, particularly in younger, fit patients. The present cohort was treated predominantly with conventional HD-MTX-, ICE-, or MPV-based regimens without rituximab, thiotepa, or BTK inhibitors, and whether subependymal enhancement retains its prognostic significance and differential association with chemoradiotherapy outcome in the context of these newer, more CNS-penetrant treatment strategies cannot be determined from the current data. Given the anatomical rationale proposed above, it is plausible that subependymal enhancement could remain a marker of poor disease control with systemic therapy alone even as regimens evolve, but this hypothesis requires direct evaluation in cohorts treated with contemporary novel agents before the observed association between subependymal enhancement and chemoradiotherapy benefit can be generalized to treatment intensification strategies, radiotherapy-inclusive or otherwise.

Several limitations of the present study warrant acknowledgment. This was a retrospective single-center study with a small sample size, and the number of patients with subependymal enhancement who received chemoradiotherapy was particularly limited. Although the observed effect sizes were large and consistent across PFS and OS, the formal interaction test did not reach significance, almost certainly reflecting inadequate statistical power. The retrospective design introduces potential selection bias, as treatment was not randomized. The reproducibility of MRI-based assessment of subependymal enhancement across different readers and institutions was not formally evaluated, and inter-rater reliability data are lacking. Importantly, chemoradiotherapy was not used in any patient treated before 2018, so treatment era and receipt of chemoradiotherapy were closely correlated (Section 3.6); although subependymal enhancement remained independently prognostic after adjustment for treatment era, the effect of chemoradiotherapy itself could not be fully disentangled from concurrent temporal changes in supportive care, staging accuracy, and referral patterns over the 15-year study period. Rituximab was not incorporated into either first-line or salvage/relapse regimens in this cohort, which predates the routine adoption of rituximab-containing induction such as the MATRix regimen; whether the differential chemoradiotherapy benefit associated with subependymal enhancement persists in the context of rituximab- or thiotepa-based regimens, or other emerging therapeutic strategies, remains to be determined. In addition, the IELSG score could only be partially reconstructed (four of five components) because CSF protein was not uniformly available in this retrospective cohort, which may limit the precision of comparisons between subependymal enhancement and existing prognostic models. Prospective validation in a larger multicenter cohort, ideally incorporating subependymal enhancement as a pre-specified stratification variable in a randomized treatment trial, is essential before these imaging biomarkers can be translated into routine clinical practice. Future studies may also explore whether machine learning–based integration of these MRI features could further refine individualized risk stratification [15], particularly as novel therapeutic targets and treatment strategies for PCNSL continue to evolve [6].

## 5. Conclusions

In this retrospective study of 50 immunocompetent patients with PCNSL, baseline MRI findings independently predicted survival outcomes and provided clinically actionable information beyond existing prognostic scoring systems. Subependymal enhancement and leptomeningeal spread were identified as independent prognostic factors for OS, with subependymal enhancement additionally serving as an independent predictor of PFS. Critically, although subependymal enhancement was associated with poor prognosis overall, patients harboring this feature who received chemoradiotherapy achieved markedly improved outcomes, compared with dismal outcomes in those treated with chemotherapy alone. No significant survival benefit from chemoradiotherapy was observed in patients without subependymal enhancement, suggesting that the benefit of radiotherapy in PCNSL may be concentrated in this specific MRI-defined subgroup.

These findings suggest that systematic assessment of subependymal enhancement and leptomeningeal spread on baseline MRI at the time of PCNSL diagnosis may complement existing clinical scoring systems and guide more individualized treatment decisions. In particular, the presence of subependymal enhancement may warrant early and deliberate incorporation of chemoradiotherapy into the first-line treatment regimen, while its absence may support the use of chemotherapy alone, thereby potentially sparing patients from the neurotoxic risks of radiotherapy without compromising survival. Prospective validation of these MRI-based biomarkers in larger multicenter cohorts is warranted before they can be adopted as standard stratification tools in clinical practice.

## Figures and Tables

**Table 1:** Baseline characteristics of primary central nervous system lymphoma patients (*n* = 50).

Characteristic	Value (N, %)
Median age, years (range)	63 (33–79)
>60 years	32 (64.0%)
Sex ratio (male:female)	0.79 (28:22)
Median F/U period, months (range)	20.6 (0.7–148.7)
Median KPS (range)	70 (30–90)
≥70	29 (58.0%)
<70	21 (42.0%)
ECOG PS	
0–1	14 (28.0%)
2	21 (42.0%)
3–4	15 (30.0%)
Serum LDH	
Normal	25 (50.0%)
Elevated	15 (30.0%)
Unknown	10 (20.0%)
MRI findings	
N of lesions	
Solitary	25 (50.0%)
Multiple	25 (50.0%)
Radiologic location	
Cortical	27 (54.0%)
Subcortical or Deep structures	23 (46.0%)
Subependymal enhancement	22 (44.0%)
Leptomeningeal spread	9 (18.0%)
Ventricular involvement	9 (18.0%)
Surgical approach	
Stereotactic biopsy	15 (30.0%)
Open biopsy	24 (48.0%)
Resection (non-GTR)	5 (10.0%)
Resection (GTR)	6 (12.0%)
First-line Tx	
HD-MTX	30 (60.0%)
ICE ± RT	8 (16.0%)
MPV ± RT	10 (20.0%)
RT alone	2 (4.0%)
Radiologic response to the first-line Tx	
CR	25 (50%)
PR	6 (12.0%)
SD	1 (2.0%)
PD	12 (24.0%)
Unknown	6 (12.0%)
Recurrence after the first-lineTx	
Yes	33 (66.0%)
No	9 (18.0%)
Unknown	8 (16.0%)
Overall survival	
Death	38 (76.0%)
Alive	11 (22.0%)
F/U loss	1 (2.0%)

N, number; F/U, follow-up; KPS, Karnofsky Performance Status; ECOG PS, Eastern Cooperative Oncology Group Performance Status; LDH, lactate dehydrogenase; MRI, magnetic resonance imaging; GTR, gross total resection; Tx, treatment; HD-MTX, high-dose methotrexate; ICE, ifosfamide-carboplatin-etoposide; RT, radiotherapy; MPV, methotrexate-procarbazine-vincristine; CR, complete response; PR, partial response; SD, stable disease; PD, progressive disease.

**Table 2:** Univariate analysis of progression-free survival (PFS) and overall survival (OS) (*n* = 50).

Variables	PFS			OS		
	Median (Months)	HR (95% CI)	*p*-Value	Median (Months)	HR (95% CI)	*p*-Value
Sex			0.547			0.895
Male (*n* = 28)	14.8	1 (ref)		30.3	1 (ref)	
Female (*n* = 22)	23.7	1.204 (0.629–2.304)		19.7	1.040 (0.561–1.926)	
Age (years)			0.227			0.798
<60 (*n* = 16)	14.8	1 (ref)		23.6	1 (ref)	
≥60 (*n* = 34)	21.3	0.689 (0.359–1.323)		22.5	0.926 (0.494–1.735)	
KPS scale			0.542			0.768
≥70 (*n* = 29)	21.1	1 (ref)		32.2	1 (ref)	
<70 (*n* = 21)	21.2	1.206 (0.628–2.316)		22.2	1.091 (0.588–2.024)	
ECOG PS			0.649			0.684
0–2 (*n* = 35)	15.1	1 (ref)		23.6	1 (ref)	
3–4 (*n* = 15)	21.2	1.159 (0.584–2.302)		22.2	0.879 (0.453–1.704)	
Serum LDH			0.097			0.952
Normal (*n* = 25)	14.7	1 (ref)		22.5	1 (ref)	
Elevated (*n* = 15)	28.4	0.545 (0.250–1.189)		35.6	0.980 (0.474–2.023)	
Number of lesions			0.793			0.206
Solitary (*n* = 25)	21.1	1 (ref)		37.7	1 (ref)	
Multiple (*n* = 25)	21.2	0.923 (0.481–1.768)		18.4	1.443 (0.783–2.661)	
Radiologic location			0.440			0.751
Cortical (*n* = 27)	14.8	1 (ref)		21.8	1 (ref)	
Subcortical or deep structures (*n* = 23)	28.4	0.788 (0.411–1.513)		32.8	0.908 (0.481–1.717)	
Subependymal enhancement			0.054 ^†^			0.014 *
No (*n* = 28)	23.7	1 (ref)		37.7	1 (ref)	
Yes (*n* = 22)	3.2	1.792 (0.934–3.437)		17	2.075 (1.089–3.954)	
Leptomeningeal spread			0.332			0.007 *
No (*n* = 41)	21.2	1 (ref)		32.2	1 (ref)	
Yes (*n* = 9)	10.6	1.502 (0.594–3.801)		13.4	2.572 (1.174–5.635)	
Ventricular involvement			0.983			0.58
No (*n* = 41)	21.1	1 (ref)		22.5	1 (ref)	
Yes (*n* = 9)	29.8	0.992 (0.446–2.204)		30.3	1.228 (0.562–2.683)	
Radiation (1st-line)			0.029 *			0.366
Non-radiation group (*n* = 35)	10.6	1 (ref)		21.8	1 (ref)	
Radiation group (*n* = 15)	31.6	0.466 (0.221–0.982)		39.8	0.731 (0.352–1.519)	
Treatment modality (1st-line)			0.303			0.430
Chemo + RT (*n* = 13)	33.5	1 (ref)	-	43.6	1 (ref)	-
Chemo-alone (*n* = 35)	10.6	2.302 (1.047–5.060)	0.024 * vs. Chemo + RT	21.8	1.655 (0.744–3.683)	0.181 vs. Chemo + RT
RT-alone (*n* = 2)	3.3	4.653 (0.400–54.155)	0.074 ^†^ vs. Chemo + RT	13.4	4.415 (0.713–27.355)	0.047 * vs. Chemo + RT

N, number; HR, hazard ratio; CI, confidence interval; KPS, Karnofsky Performance Status; ECOG, Eastern Cooperative Oncology Group; LDH, lactate dehydrogenase; RT, radiotherapy; ref, reference; Chemo, chemotherapy. * *p* < 0.05; † borderline (*p* < 0.10).

**Table 3:** Multivariate Cox proportional hazards analysis of progression-free survival (PFS) and overall survival (OS) (*n* = 50).

Variables	PFS			OS		
	HR	95% CI	*p*-Value	HR	95% CI	*p*-Value
Subependymal enhancement	2.447	1.164–5.145	0.018 *	2.607	1.279–5.313	0.008 *
Leptomeningeal spread	1.427	0.503–4.051	0.504	3.418	1.473–7.932	0.004 *
Chemoradiotherapy	0.293	0.115–0.741	0.010 *	0.431	0.174–1.071	0.070 †
Concordance index	0.725			0.672		
LR test *p*-value	0.006			0.001		

N, number; HR, hazard ratio; CI, confidence interval; LR, likelihood ratio. * *p* < 0.05; † *p* < 0.10 (borderline significance). Reference categories: subependymal enhancement No, leptomeningeal spread No, chemoradiotherapy No (chemotherapy alone or radiotherapy alone).

## Data Availability

The data presented in this study are available on request from the corresponding author due to institutional restrictions on patient privacy.

## Supplementary Materials

The following supporting information can be downloaded at: https://www.mdpi.com/article/10.3390/cancers18182906/s1, Table S1. Per-patient first-line chemotherapy and radiotherapy regimen details (*n* = 50).

## Abbreviations

The following abbreviations are used in this manuscript:

PCNSL	Primary central nervous system lymphoma
MRI	Magnetic resonance imaging
PFS	Progression-free survival
OS	Overall survival
HD-MTX	High-dose methotrexate
WBRT	Whole-brain radiotherapy
IELSG	International Extranodal Lymphoma Study Group
MSKCC	Memorial Sloan-Kettering Cancer Center
CSF	Cerebrospinal fluid
DLBCL	Diffuse large B-cell lymphoma
KPS	Karnofsky Performance Status
ECOG	Eastern Cooperative Oncology Group
LDH	Lactate dehydrogenase
HR	Hazard ratio
CI	Confidence interval
CR	Complete response
PR	Partial response
SD	Stable disease
PD	Progressive disease
ORR	Overall response rate
RT	Radiotherapy
