# Supplementary material for: Prognostic Value of Subependymal Enhancement on Baseline MRI in Primary Central Nervous System Lymphoma
Source: Cancers (Basel). 2026 Sep 8;18(18):2906. doi: 10.3390/cancers18182906 (PMC13604352; doi:10.3390/cancers18182906)
Supplement: Supplementary file 1 [file cancers-18-02906-s001.zip › cancers-4524020-supplementary.pdf]

# Supplementary Materials

**Table S1. Per-patient first-line chemotherapy and radiotherapy regimen details ($n$ = 50).**

| No. | First-Line Regimen (Category) | Regimen Detail (Dose / Cycles / RT Dose-Fractionation, as Documented) |
|---|---|---|
| 1 | HD-MTX | HD-MTX 8.0g/m$^2$ (induction; 3 cycles), 3.5g/m$^2$ (consolidation; 6 cycles) |
| 2 | HD-MTX | HD-MTX 8.0g/m$^2$ (induction; 3 cycles) |
| 3 | HD-MTX | HD-MTX 8.0g/m$^2$ (induction; 3 cycles) |
| 4 | HD-MTX | HD-MTX 8.0g/m$^2$ (induction; 1 cycle) |
| 5 | HD-MTX | HD-MTX 8.0g/m$^2$ (induction; 5 cycles) |
| 6 | HD-MTX | HD-MTX 8.0g/m$^2$ (induction; 3 cycles), 3.5g/m$^2$ (consolidation; 6 cycles) |
| 7 | HD-MTX | HD-MTX 8.0g/m$^2$ (induction; 3 cycles) |
| 8 | HD-MTX | HD-MTX 8.0g/m$^2$ (induction; 4 cycles), 3.5g/m$^2$ (consolidation; 4 cycles) |
| 9 | HD-MTX | HD-MTX 8.0g/m$^2$ (induction; 3 cycles), 3.5g/m$^2$ (consolidation; 4 cycles) |
| 10 | HD-MTX | HD-MTX 8.0g/m$^2$ (induction; 5 cycles) |
| 11 | HD-MTX | HD-MTX 8.0g/m$^2$ (induction; 4 cycles), 3.5g/m$^2$ (consolidation; 1 cycle) |
| 12 | HD-MTX | HD-MTX 8.0g/m$^2$ (induction; 2 cycles) |
| 13 | HD-MTX | HD-MTX 8.0g/m$^2$ (induction; 3 cycles), 3.5g/m$^2$ (consolidation; 6 cycles) |
| 14 | HD-MTX | HD-MTX 8.0g/m$^2$ (induction; 3 cycles) |
| 15 | HD-MTX | HD-MTX 3.5g/m$^2$ (induction; 2 cycles) |
| 16 | HD-MTX | HD-MTX 8.0g/m$^2$ (induction; 2 cycles) |
| 17 | HD-MTX | HD-MTX 4.0g/m$^2$ (induction; 1 cycle) |
| 18 | HD-MTX | HD-MTX 8.0g/m$^2$ (induction; 3 cycles), 3.5g/m$^2$ (consolidation; 6 cycles) |
| 19 | HD-MTX | HD-MTX 8.0g/m$^2$ (induction; 3 cycles) |
| 20 | HD-MTX | HD-MTX 8.0g/m$^2$ (induction; 3 cycles), 3.5g/m$^2$ (consolidation; 6 cycles) |
| 21 | HD-MTX | HD-MTX 8.0g/m$^2$ (induction; 3 cycles), 3.5g/m$^2$ (consolidation; 2 cycles) |
| 22 | HD-MTX | HD-MTX 8.0g/m$^2$ (induction; 2 cycles) |
| 23 | HD-MTX | HD-MTX 8.0g/m$^2$ (induction; 3 cycles) |
| 24 | HD-MTX | HD-MTX 8.0g/m$^2$ (induction; 3 cycles), 3.5g/m$^2$ (consolidation; 6 cycles) |
| 25 | HD-MTX | HD-MTX 8.0g/m$^2$ (induction; 5 cycles), 3.5g/m$^2$ (consolidation; 5 cycles) |
| 26 | HD-MTX | HD-MTX 8.0g/m$^2$ (induction; 4 cycles) |
| 27 | HD-MTX | HD-MTX 8.0g/m$^2$ (induction; 3 cycles), 3.5g/m$^2$ (consolidation; 6 cycles) |
| 28 | HD-MTX | HD-MTX 8.0g/m$^2$ (induction; 1 cycle) |
| 29 | HD-MTX | HD-MTX 8.0g/m$^2$ (induction; 3 cycles), 3.5g/m$^2$ (consolidation; 2 cycles) |
| 30 | HD-MTX | HD-MTX 8.0g/m$^2$ (induction; 3 cycles), 3.5g/m$^2$ (consolidation; 2 cycles) |
| 31 | ICE+RT | ICE (6 cycles) + RTx (25.2Gy/15fx) |
| 32 | ICE | ICE (4 cycles) |
| 33 | ICE+RT | ICE (6 cycles) + RTx (45Gy/25fx) |
| 34 | ICE+RT | ICE (6 cycles) + WBRT 27Gy/15fx |
| 35 | ICE+RT | ICE (3 cycles) + RTx (50.6Gy/27fx) |
| 36 | ICE+RT | ICE (6 cycles) + WBRT (23.4Gy/13fx) |
| 37 | RT | RTx [WBRT(30Gy/15fx) + boost RTx (15Gy/5fx)] |
| 38 | ICE | ICE (2 cycles) |
| 39 | ICE | ICE (2 cycles) |
| 40 | MPV+RT | MPV (6 cycles) + RTx [(WBRT 30Gy/15fx) + boost RTx (20Gy/10fx)] |
| 41 | RT | RTx [WBRT (36Gy/18fx) + boost RTx (14.4Gy/8fx)] |
| 42 | MPV+RT | MPV (5 cycles) + RTx [(WBRTx (30.6Gy/17fx) + boost RTx (19.8/11fx)] |

| | | |
|---|---|---|
| 43 | MPV+RT | MPV (1 cycle) + WBRT (46.6Gy/35fx) |
| 44 | MPV+RT | MPV (6 cycles) + RTx [(WBRTx (30.6/18fx) + boost RTx (16.8/8fx)] |
| 45 | MPV | MPV (6 cylces) |
| 46 | MPV+RT | MPV (1 cycle) + RTx [WBRT (30 Gy/15fx) + boost RTx (20 Gy/10fx)] |
| 47 | MPV | MPV (1 cycle) |
| 48 | MPV+RT | MPV (6 cycles) + WBRT (25.2 Gy/14 fx) |
| 49 | MPV+RT | MPV (6 cycles) + RTx [WBRT (25.2Gy/14fx) +boost RTx (22 Gy/11 fx)] |
| 50 | MPV+RT | MPV (4 cycles) + RTx [WBRT (30.6 Gy/17fx) + boost RTx (14.4 Gy/8 fx)] |

Patients are listed in de-identified sequential order (No. 1–50); this order does not correspond to registry or enrollment order. HD-MTX doses are per cycle. Radiotherapy regimens for patients receiving ICE, MPV, ICE+RT, MPV+RT, or RT alone are reported as documented, including whole-brain radiotherapy (WBRT) dose/fractionation and consolidative boost dose/fractionation where applicable. HD-MTX, high-dose methotrexate; ICE, ifosfamide-carboplatin-etoposide; MPV, methotrexate-procarbazine-vincristine; RT, radiotherapy; WBRT, whole-brain radiotherapy; fx, fractions.
